# Neonatal diabetes mellitus: a disease linked to multiple mechanisms

**DOI:** 10.1186/1750-1172-2-12

**Published:** 2007-03-09

**Authors:** Michel Polak, Hélène Cavé

**Affiliations:** 1 Faculty of medicine Paris René Descartes, Paediatric endocrinology and INSERM U845, Hôpital Necker-Enfants Malades, 149 rue de Sèvres, Paris, France; 2 Département de Génétique, Hôpital Robert Debré, 48 Boulevard Sérurier, 75019 Paris, France

## Abstract

Transient (TNDM) and Permanent (PNDM) Neonatal Diabetes Mellitus are rare conditions occurring in 1:300,000–400,000 live births. TNDM infants develop diabetes in the first few weeks of life but go into remission in a few months, with possible relapse to a permanent diabetes state usually around adolescence or as adults. The pancreatic dysfunction in this condition may be maintained throughout life, with relapse initiated at times of metabolic stress such as puberty or pregnancy. In PNDM, insulin secretory failure occurs in the late fetal or early post-natal period and does not go into remission. Patients with TNDM are more likely to have intrauterine growth retardation and less likely to develop ketoacidosis than patients with PNDM. In TNDM, patients are younger at the diagnosis of diabetes and have lower initial insulin requirements. Considerable overlap occurs between the two groups, so that TNDM cannot be distinguished from PNDM based on clinical features. Very early onset diabetes mellitus seems to be unrelated to autoimmunity in most instances. A number of conditions are associated with PNDM, some of which have been elucidated at the molecular level. Among these, the very recently elucidated mutations in the *KCNJ11 *and *ABCC8 *genes, encoding the Kir6.2 and SUR1 subunit of the pancreatic K_ATP _channel involved in regulation of insulin secretion, account for one third to half of the PNDM cases. Molecular analysis of chromosome 6 anomalies (found in more than 60% in TNDM), and the *KCNJ11 *and *ABCC8 *genes encoding Kir6.2 and SUR1, provides a tool to identify TNDM from PNDM in the neonatal period. This analysis also has potentially important therapeutic consequences leading to transfer some patients, those with mutations in *KCNJ11 *and *ABCC8 *genes, from insulin therapy to sulfonylureas. Recurrent diabetes is common in patients with "transient" neonatal diabetes mellitus and, consequently, prolonged follow-up is imperative. Realizing how difficult it is to take care of a child of this age with diabetes mellitus should prompt clinicians to transfer these children to specialized centers. Insulin therapy and high caloric intake are the basis of the treatment. Insulin pump may offer an interesting therapeutic tool in this age group in experienced hands.

## Definition, forms and epidemiology

Neonatal diabetes mellitus (NDM) is a rare (1:300,000–400,000 newborns) but potentially devastating metabolic disorder characterized by hyperglycemia combined with low levels of insulin. Two main groups have been recognized on clinical grounds, transient NDM (TNDM) and permanent NDM (PNDM), which differ in the duration of insulin dependence early in the disease. TNDM is a developmental disorder of insulin production that resolves postnatally and represents 50% to 60% of cases of neonatal diabetes. There are no clinical features that can predict whether a neonate with diabetes (but no other dysmorphic features) will eventually have permanent or transient disease. Recently, advances have been made in the understanding of the molecular mechanisms of pancreatic development that are relevant to PNDM and TNDM (Table 1). This review focuses on the clinical features and the molecular causes of these varied conditions. It also underlines how the molecular understanding of some forms of neonatal diabetes led to transfer patients from insulin injections to oral sulfonylureas, providing a spectacular example of a pharmacogenomic approach.

**Table 1:** Etiologies of neonatal diabetes

**Transient neonatal diabetes mellitus**	**Permanent neonatal diabetes mellitus**
• Chromosome 6 anomalies detected	• Heterozygous activating mutation in *KCNJ11 gene *and in *ABCC8 gene *(Kir6.2 and SUR1 subunits of the pancreatic K_ATP _channel)
- paternal duplications	
- paternal isodisomy	
- Methylation defect	
• *ABCC8 *(SUR1) and rarely *KCNJ11 *(Kir6.2) mutations	• IPEX syndrome: diffuse autoimmunity • Mitochondrial disease • Severe pancreatic hypoplasia associated with IPF1 (*PDX1*) mutation • Homozygous glucokinase mutation: insensitivity to glucose • Associated with epiphyseal dysplasia: Wolcott Rallison syndrome • Possibly associated with enterovirus infection • Association with cerebellar hypoplasia and *PTF1A *mutation • Association with hypothyroidism, glaucoma and *GLIS3 *mutation

## I. Clinical description

Neonatal diabetes mellitus presents as hyperglycemia, failure to thrive and, in some cases, dehydration and ketoacidosis, which may be severe with coma in a child within the first months of life. Insulin production is inadequate with a low blood level in comparison with the hyperglycemia, and therefore exogenous insulin therapy is required.

### A. Clinical description of TNDM

TNDM is a developmental disorder of insulin production that resolves postnatally. TNDM represents 50% to 60% of cases of neonatal diabetes [1,2]. Intrauterine growth retardation (IUGR) is usually present. The high rate of IUGR is in keeping with the crucial role of insulin in fetal growth, especially during the last trimester of pregnancy. Hyperglycemia, failure to thrive and, in some cases, dehydration occur after birth. Insulin production is inadequate, requiring exogenous insulin therapy. Tests are negative for anti-islet antibodies and for HLA class II haplotypes conferring susceptibility to type 1 diabetes [2]. A defect in cell maturation has been suggested [3]. Interestingly, exocrine pancreatic insufficiency is present in only a few patients [4]. However, the cellular basis of TNDM remains unknown. Most patients recover within a year, but a few have persistent glucose intolerance and/or recurrence of diabetes in late childhood or adulthood. Although these recurrences are usually consistent with non-autoimmune type 1 diabetes, whether they are ascribable to insulin deficiency and/or insulin resistance remains unclear [1,2,5]. Indeed, a permanent hyperglycemia requiring insulin therapy developed in five of the seven TNDM patients who were older than 8 years of age in a French cohort [6]. Similarly, in another large cohort of TNDM patients, diabetes mellitus recurred in 11 out of 18 patients older than 4 years of age [7]. Thus, the "transient" form of the disease is probably a permanent **β**-cell defect with variable expression during growth and development. A major factor in the onset of recurrent diabetes is probably puberty, which is associated with significant insulin resistance.

We examined derived indices of pancreatic **β**-cell function, peripheral insulin sensitivity and the pancreatic response to intravenous glucose loading in children with a previous history of transient neonatal diabetes currently in remission repeated after a period of two years [8]. One child had a sub-normal insulin secretory response to intravenous glucose that remained abnormal two years later. The other children had relatively normal or entirely normal responses over two years. Measures of **β**-cell function and insulin sensitivity in the fasting state showed comparable results to those obtained from normal controls [8]. We concluded that the majority of children with TNDM in remission have no evidence of **β**-cell dysfunction or insulin resistance in the fasting state. Measures of insulin response to intravenous glucose loading are often normal but suggestive of future recurrence if profoundly abnormal [8].

Table 2 compares several clinical features of TNDM and PNDM in the French cohort (n = 50) [9].

**Table 2:** Comparison of several features in PNDM and TNDM cases in the French cohort (n = 50) (adapted from [9]).

	**PNDM *n *= 21**	**TNDM *n *= 29**	***P *value**
Gestational age (weeks)	39.2 ± 1.6	38.2 ± 2.2	*P = *0.15
Birth weight (g)	2497 ± 690	1987 ± 510	*P *< 0.006
Birth length (cm)	47.5 ± 2.4	44.3 ± 3.4	*P *< 0.006
Head circumference (cm)	33 ± 1.9	31.5 ± 1.8	*P *< 0.02
Intrauterine growth retardation	*n *= 7/19 36%	*n *= 20/27 74%	*P *< 0.03
Median age at diagnosis (days) (range)*	27 (1–127)	6 (1–81)	*P *< 0.01
Initial insulin dose (unit/kg/day)	1.4 ± 1.2	0.6 ± 0.25	*P *< 0.006

* Given the non-Gaussian distribution of age in the study population, the nonparametric Mann-Whitney U-test was used to compare age in the two groups.

### B. Clinical description of PNDM

Permanent neonatal diabetes mellitus is less common than the transient form of the condition. By definition, diabetes develops in the neonatal period and does not go into remission. There are no clinical features that can predict whether a neonate with diabetes but no other dysmorphic features will eventually have permanent or transient disease, although cases with the permanent form do not always have IUGR as is universally seen in the transient 6q phenotype (see below) (Table 2) [6,7]. Diabetes in infancy is nearly always unrelated to classical type 1 diabetes [10]. In an Italian study conducted in all infants developing diabetes before the age of one year, a clear difference was demonstrated between those infants developing diabetes before the age of 180 days and those after. The children developing diabetes early had a very high presence of "protective" HLA alleles against classical type 1 diabetes (76% with 0 or 1 susceptibility heterodimers), compared to only 12% in the late (>180 days) onset group [11]. In addition, autoimmune markers were far less prevalent in the early onset group of children compared to children with late onset diabetes (15% *vs *65% if onset after 180 days).

## II. Mechanisms

### A. Molecular mechanisms of TNDM

**Figure 1:**
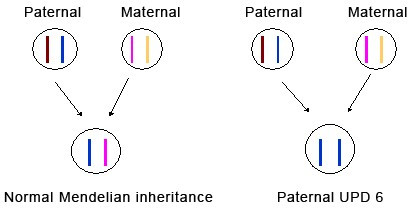
Schematic representation of paternal uniparental disomy of chromosome 6. In cases of paternal UPD 6, two alleles are inherited from the father.

**Figure 2:**
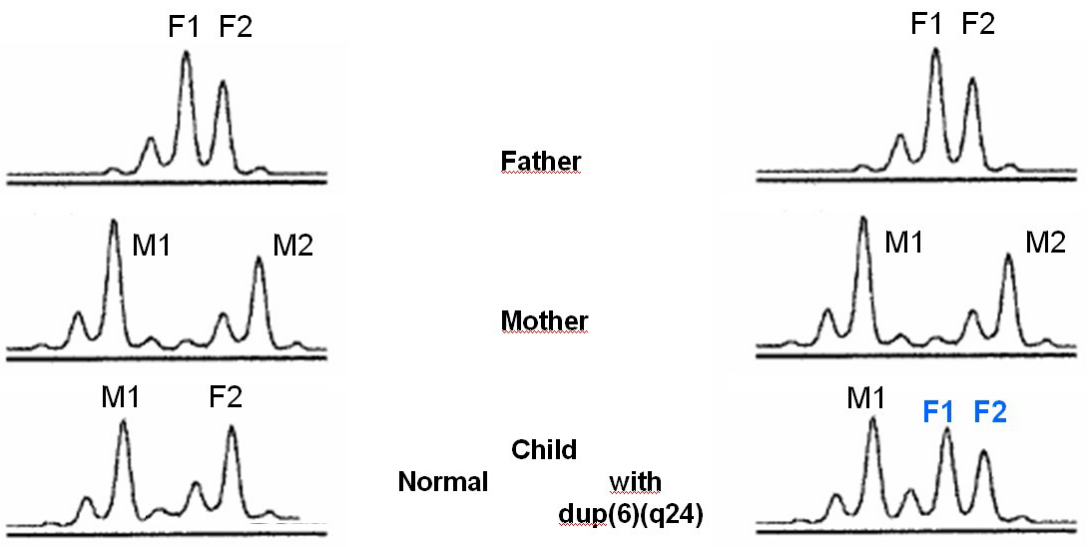
**Dup(6)(q24): Inheritance of 2 paternal alleles (F1+F2)**. M (mother), F (father).

**Figure 3:**
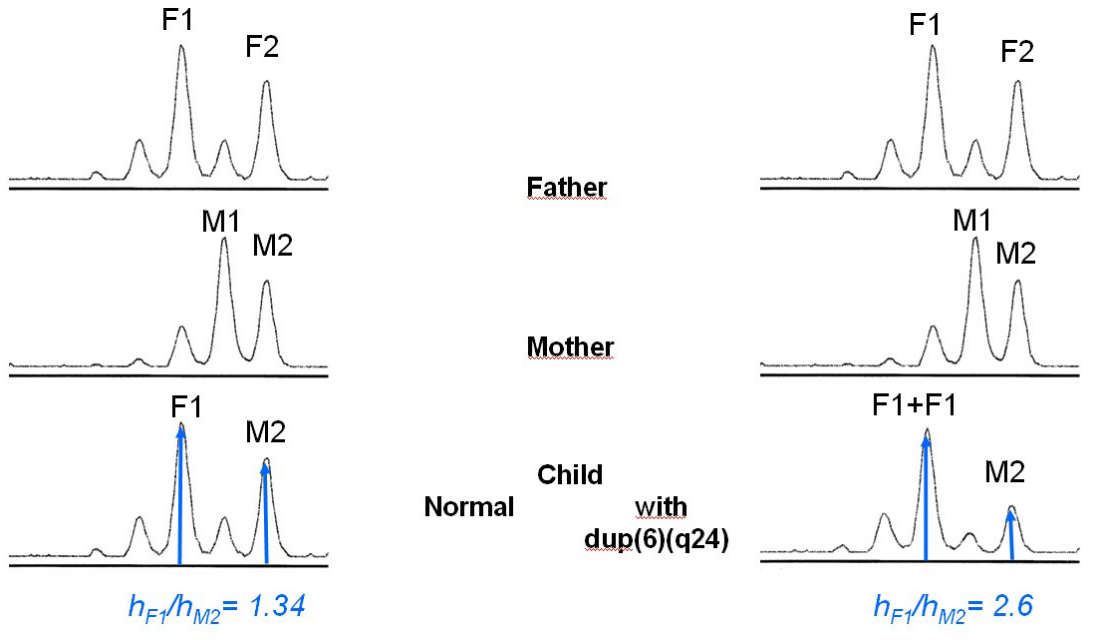
**Dup(6)(q24): Increase dosage of one paternal allele (F1)**. M (mother), F (father), h (peak height).

**Figure 4:**
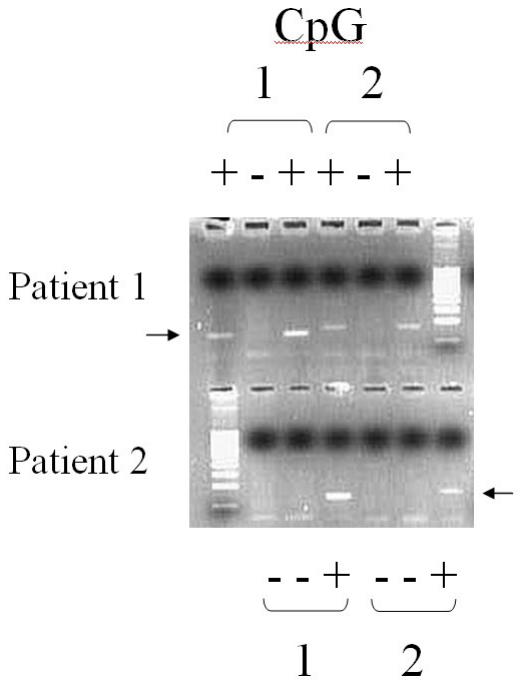
PCR after digestion of the DNA with a methylation sensitive enzyme. In the case of a methylation defect, one loses the amplicon normally present and due to the maternal methylated allele.

TNDM is usually sporadic, but paternal transmission has been documented in about one-third of reported patients, some of whom had non-diabetic fathers [1,12]. Paternal isodisomy of chromosome 6 has been demonstrated in several unrelated patients with TNDM (Figure 1). Other patients had partial duplications of the long arm of the paternal chromosome 6 [13,14]. When these unbalanced duplications are inherited within families. TNDM arises only if the duplication is inherited from the father, suggesting a disorder of imprinting. More recently, a region in which methylation differs between the maternal and the paternal chromosome 6 has been identified [14]. Abnormal methylation patterns have been documented in some TNDM patients without other chromosome 6 abnormalities (Table 1) (Figures 2, 3, 4) [15]. These observations strongly suggest that TNDM may result from over-expression of an imprinted gene located on chromosome 6q24 and displaying paternal expression. Two paternally expressed genes are located in the region and, therefore, have been considered candidate genes for the disease: one is the gene encoding transcription factor ZAC (*LOT1*, *PLAGL-1*) that regulates cell cycle arrest and apoptosis and also the Pituitary Adenylate Cyclase Activating Polypeptide Receptor 1 (PACAP1) being a potent insulin secretagogue, and the other is the *HYMAI *gene, whose function is unknown [16]. No other loci have been implicated in TNDM to date. Recently, an animal model of TNDM has been generated by the insertion of the human TNDM locus into a mouse. Mice over-expressing the TNDM locus display many, but not all, of the features of human TNDM [17]. Paternal transmission leads to neonatal hyperglycemia and an increased tendency for diabetes in later life. Interestingly, over-expression of the TNDM locus reduces the expression of the key transcription factor PDX-1 in the embryonic pancreas of these mice [17]. Nevertheless, the precise link between those genetic anomalies and the insulin secreting cell impaired function remained to be established.

### B. Molecular mechanisms of PNDM

#### B1. The insulin cell potassium channel (Kir6.2/SUR1) (Figures 5 and 6)

**Figure 5:**
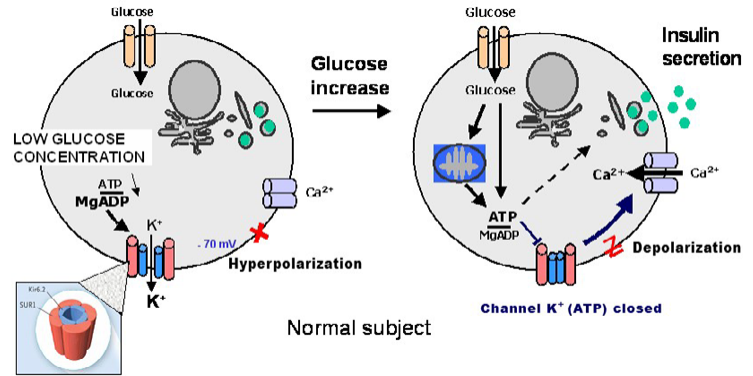
Insulin secretion in relation to the potassium channel encoded for by *KCNJ11 *and *ABCC8 *in a normal subject.

**Figure 6:**
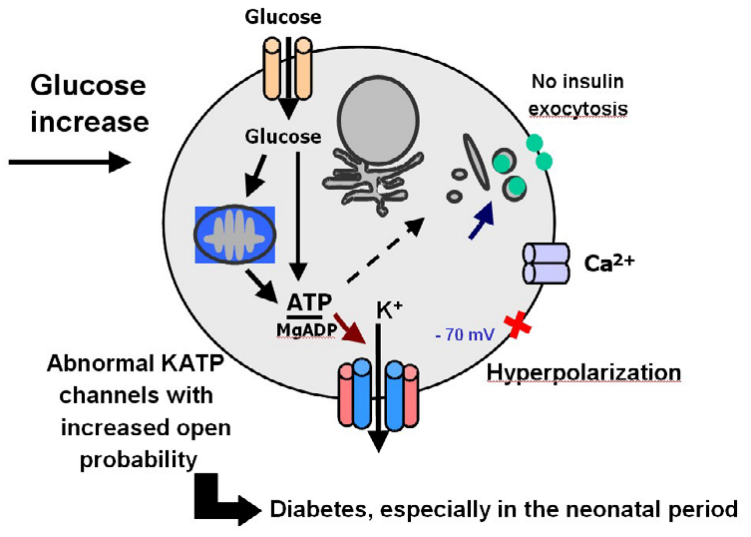
Heterozygous activating mutations in the *KCNJ11 *and *ABCC8 *genes encoding the Kir6.2 and SUR1 subunit of the pancreatic beta-cell potassium ATP (KATP) channel cause PNDM. This mutations led to an increase probability of opening of the potassium channel therefore preventing any activation of the voltage dependent calcium channel and any glucose induced insulin secretion.

Mutations in *KCNJ11 *encoding the Kir6.2 subunit of the pancreatic ATP-sensitive potassium channels (K_ATP_) couple cell metabolism to electrical activity by regulating potassium movement across the membrane. These channels are made up of an octameric complex with two kind of subunits: four regulatory sulfonylurea receptors (SUR) embracing four pore forming inwardly rectifying potassium channels (Kir). A 1:1 SUR1:KIR6.2 stochiometry is both necessary and sufficient for assembly of active K_ATP _channels. SUR, a member of the ABC transporter family, originates from two separate genes and therefore occurs in several spliced isoforms. SUR1 is found in the pancreatic **β**-cells and neurons, whereas SUR2A is in heart cells and SUR2B in smooth muscle. The Kir6.2 subunit forms the channel pore in the majority of tissues such as pancreatic **β**-cells, brain, heart and skeletal muscles, while Kir6.1 can be found in smooth vascular muscle and astrocytes. These different channel forms have different pore properties and adenine nucleotide sensitivity.

Recently, Gloyn *et al*. reported in an ethnically diverse patient cohort that six heterozygous activating mutations in the *KCNJ11 *gene encoding the Kir6.2 subunit of the pancreatic **β**-cell potassium ATP (K_ATP_) channel caused PNDM in ten probands [18]. Some of these mutations are also associated with developmental delay, muscle weakness and epilepsy [18]. We screened the *KCNJ11 *gene for mutations in patients with PNDM recruited through the French network for the study of neonatal diabetes. Seventeen at-term babies with a median age of 64 days at diagnosis of diabetes (range 1–260) were included [19]. We identified seven heterozygous non-synonymous mutations in nine patients: three (V59M, R201C, R201H) were already described by Gloyn *et al*.; the four novel mutations resulted in amino acid changes of Kir6.2 at positions F35L, G53N, E322K, Y330C. More patients with a Kir6.2 mutation (6/9) were reported to have a smaller birth weight than those without the mutation (2/8), confirming the findings of Gloyn *et al*. [18,19]. These mutations lead to a permanent opening of the potassium channel, therefore preventing any activation of the voltage-dependent calcium channel and any glucose-induced insulin secretion (Figures 5 and 6). Although Kir6.2 mutation carriers do not represent a phenotypically specific form of PNDM, an impaired function of Kir6.2 is associated with *in utero *insulin secretory insufficiency and growth retardation. In conclusion, Kir6.2 mutations are a common cause, from one third to a half of PNDM in Caucasians. This work has opened new avenues for the work-up and treatment of those patients (see below) [20].

##### Mutations in *ABCC8 *encoding the SUR1 subunit of the pancreatic K_ATP _channel

As explained above, the ATP-gated potassium (K_ATP_) channel (composed of SUR1 and K_IR_6.2 proteins) is a key regulator of insulin release. It is inhibited by the binding of adenine nucleotides to K_IR_6.2 leading to closing of the channel, and activated by nucleotide binding and/or hydrolysis on SUR1 leading to opening of the channel. The balance of these opposing actions determines the low open channel probability, P_0_, which controls the excitability of pancreatic **β**-cells. We hypothesized that activating mutations in *ABCC8 *which encodes SUR1 cause neonatal diabetes. We therefore screened the 39 exons of *ABCC8 *in 34 patients diagnosed with permanent or transient neonatal diabetes (ND) of unknown origin in a case series of 73 ND patients [21]. We assayed the electrophysiological activity of mutant and wild-type K_ATP _channels. We identified seven missense mutations in nine patients. Four mutations were familial and showed vertical transmission with neonatal and adult onset diabetes; the remaining mutations were *de novo *and not found in more than 300 non-diabetic or early onset diabetic subjects of similar genetic background. Mutant channels in intact cells and in physiologic concentrations of MgATP had a markedly higher P_0 _compared with wild type channels. These over-active channels retained sulfonylurea sensitivity and treatment with sulfonylureas achieved euglycemia. Dominant mutations in *ABCC8 *account for 12% (9/73) of our ND cases. Diabetes results from a novel mechanism whereby the basal Mg-nucleotide-dependent stimulatory action of SUR1 on the K_IR _pore is elevated and block by sulfonylureas is preserved [21] (Figures 5 and 6).

#### B2. "Transient" and permanent neonatal diabetes: a common molecular mechanism?

The clinical difference between a transient and a permanent form of neonatal diabetes is not always accounted for by a different molecular mechanism. The methylation anomalies (chromosome 6q anomalies) have not so far been found to be associated with permanent neonatal diabetes. On the contrary, mutations in the SUR1 and Kir6.2 subunit have been found in association with transient and permanent neonatal diabetes. Of note is that mutations in Kir6.2 have been found mostly in association with very early forms of diabetes, usually before 6 months of age, whereas the phenotypic variability of SUR1 mutations is broader [21,22]. Indeed, mutations in SUR1 can be linked to ketoacidosis in a newborn, as well as to *bona fide *type 2 diabetes in a young adult [21].

## III. Syndromes associated with PNDM

A number of discrete clinical syndromes have been identified as associated with PNDM.

### A. Pancreas agenesis and the *Insulin Promoter Factor-1 *(*IPF1*) gene

The first patient described was a child with pancreatic agenesis and marked endocrine and exocrine failure. Insulin Promoter Factor-1 appeared to be a good candidate for pancreatic agenesis, given its role as a master control of exocrine and endocrine pancreatic development identified from studies of gene disruption [23], and later, as a regulator of insulin and somatostatin gene expression [24]. The child was homozygous for a single nucleotide deletion within codon 63 of *IPF-1 *(Pro63fsdelC) [25].

Furthermore, eight individuals from six generations with early onset diabetes akin to type 2 diabetes have been identified within the extended family. These were identified as heterozygotes for the same mutation, with the mutant truncated isoform of *IPF-1 *acting as a dominant negative inhibitor of wild type *IPF-1 *activity [26]. The illness resulting from heterozygosity was reassigned as Maturity Onset Diabetes of the Young (MODY) 4. Additional studies have also identified that less severe *IPF-1 *mutations can cause autosomal dominant late onset forms of type 2 diabetes that account for around 6% of a French cohort of multiplex type 2 diabetic families.

### B. Anomalies at the homozygous state in the *Glucokinase gene*

MODY 2 is caused by mutations in the *glucokinase *gene and usually leads to mild hyperglycemia in affected individuals [27]. Glucokinase is a key regulator of glucose metabolism in islet cells, controlling the levels of insulin secretion. However, within two families (one Norwegian and one Italian) with multiple forms of diabetes in their pedigree, two infants with classical PNDM (presenting on day one) have been identified. They were homozygous for missense mutations within the *glucokinase *gene; this rendered them completely deficient in glycolytic activity, whilst their apparently consanguineous and mild to moderately glucose intolerant parents were heterozygous for the same mutations [28]. A search for further permanent neonatal diabetes cases caused by homozygosity in *glucokinase *mutations both in British and French cohorts (total number 18) has yielded none suggesting that this is unlikely to be a major cause of PNDM [29,30]. However, we would recommend that if there is a history of gestational diabetes, testing for fasting glucose levels in both parents is needed. If both parents have mild glucose intolerance, a screen for *glucokinase *mutations is then warranted.

### C. IPEX syndrome and *FOXP3 gene*

A number of authors have reported an X-linked syndrome with a combination of exfoliative dermatitis, intractable diarrhea with villous atrophy, hemolytic anemia, autoimmune thyroid disease and neonatal onset diabetes. Most children die in the first year of life with overwhelming sepsis [31]. In some of these cases, agenesis of the islets of Langerhans has been described [32]. The idea of an autoimmune basis to this disease was strengthened by the apparent success of cyclosporin A therapy in improving the condition of one or two cases [33]. Identification of Glutamic Acid Decarboxylase (GAD) antibodies in a patient with this condition prior to bone marrow transplantation suggests that this may be a form of neonatal diabetes with an autoimmune origin. Bone marrow transplantation conditioning (anti-T lymphocyte globulin, busulfan and cyclophosphamide) led to disappearance of the diabetes a week before transplantation. Subsequently, the diarrhea resolved, as did the dermatitis. The patient remained in remission for two years prior to the development of a hemophagocytic syndrome that proved fatal [34]. The mutation in this condition lies in the *FOXP3 *gene that encodes a forkhead domain-containing protein [35]. The scurfy mouse with a frame-shift mutation in *Foxp3 *is characterized by over-proliferation of CD4+/CD8-T lymphocytes with multi-organ infiltration. The males die 15–25 days after birth [9]. It has now been demonstrated that the protein product 'scurfin' is essential for normal immune homeostasis.

### D. Wolcott-Rallison and *EIF2AK3 *gene

Wolcott-Rallison syndrome is an autosomal recessive disorder characterized by infancy onset (often within the neonatal period) diabetes associated with a spondyloepiphyseal dysplasia. In addition, there is a constellation of other features such as hepatomegaly, mental retardation, renal failure and early death [36]. In 2000, Delepine *et al*. used two consanguineous families to map the condition to the locus 2p12 [37]. Within this locus lays the gene *EIF2AK3 *that is highly expressed in islet cells and acts as a regulator of protein synthesis. Proteins and insulin are manufactured in the endoplasmic reticulum (ER). In response to environmental stresses, cells down regulate protein synthesis by phosphorylation of the alpha subunit of eukaryotic translation initiation factor-2 (eif2-alpha) by eukaryotic translation initiation factor-2 kinase3 (*EIF2AK3*). Mal-folded proteins in the ER inhibit further translation initiation mediated by increased phosphorylation of eif2-alpha. A targeted mutation of the mouse *Eif2ak3 *gene (PERK) led to an accumulation of mal-folded proteins in the ER, with resultant abnormally elevated protein synthesis and increased stress on ER folding machinery [38]. PERK is highly expressed in mouse pancreas. The PERK knock-out mouse demonstrates normal pancreatic endocrine and exocrine development. However, postnatally the mice develop ER distension, accompanied by increased cell death and progressive diabetes mellitus and pancreatic exocrine failure [39]. Further analysis within the consanguineous Wolcott-Rallison families confirmed frameshift or amino-acid substitution mutations occurring in *EIF2AK3 *segregating with the disorder in each family [37].

### E. Other syndromes with PNDM

In 1992, Christen *et al*. described two boys with X-linked phosphoribosyl-ATP pyrophosphatase hyperactivity who became diabetic on day one of life. Glucose intolerance persisted throughout life, although there were periods off insulin, as the children grew older. Both boys had other major problems including mental retardation, ataxia and progressive axonal neuropathy. The mother also had hyperuricemia (gout) and glucose intolerance with a history of gestational diabetes [40].

In 1994, Yorifuji *et al*. described a condition of neonatal diabetes associated with severe hypoplasia of the pancreas (only head and uncus present) and congenital cyanotic heart disease in a single family with apparent autosomal dominant inheritance. Not all the cases developed diabetes as a neonate, the timing is probably related to the size of remaining pancreatic tissue [41].

Recently, another severe syndrome was described in which three members of a consanguineous family developed neonatal diabetes and cerebellar hypoplasia. An autosomal recessive inheritance pattern was suggested. The infants all died within a few months of birth from a combination of metabolic dysfunction, respiratory compromise and sepsis [42]. Interestingly, there are a number of specific transcriptional activators which regulate gene expression present in both **β**-cells and neuronal tissues: dysfunction of any of these might explain the two components of this syndrome [43]. Indeed, this syndrome was found to be linked to mutations in the PTF1A transcription factor, a major gene involved in pancreatic development and also expressed in the cerebellum [44]. These patients have pancreatic hypoplasia associated with microcephaly linked to cerebellar hypoplasia [44].

Very recently, mutations in Glis3 (another transcription factor) were found to explain a syndrome which associated neonatal diabetes, hypothyroidism, congenital glaucoma, kidney cysts and hepatic fibrosis [45].

A single case report has suggested that maternal enterovirus (echovirus 6) infection in pregnancy (end of first trimester) can lead to autoimmune, neonatal onset diabetes with the presence of anti-insulin and glutamic acid decarboxylase antibodies at birth or very soon after birth. In this female child (ruling out IPEX), the pancreas was very hypoplastic and the authors suggested a role for maternally transmitted enterovirus either by direct influence on pancreatic organogenesis or through aggressive **β**-cells targeted autoimmune attack [46].

It is worth mentioning that neonatal diabetes may also exist in the context of a mitochondrial disorder [6]. It is usually associated with other organ dysfunction, which may be recognized after the diabetes mellitus diagnosis.

## IV. Clinical and biological diagnosis

### A. Diagnosis of the "transient" or permanent nature of the neonatal diabetes

The following conclusions can be drawn from current knowledge on neonatal diabetes mellitus: 1) Patients with TNDM are more likely to have intrauterine growth retardation and less likely to develop ketoacidosis than patients with PNDM; 2) TNDM patients are younger at the age of diagnosis of diabetes and have lower initial insulin requirements; 3) Considerable overlap occurs between the two groups, so that TNDM cannot be distinguished from PNDM based on clinical features; 4) Very early onset diabetes mellitus seems to be unrelated to autoimmunity in most instances; 5) Recurrent diabetes is common in patients with "transient" neonatal diabetes mellitus and, consequently, prolonged follow-up is imperative; 6) Molecular analysis of chromosome 6 anomalies, the *KCNJ11 *and *ABCC8 *genes (encoding Kir6.2 and SUR1 respectively) provide a tool for identifying transient from permanent neonatal diabetes mellitus in the neonatal period; 7) About 50% of the PNDM cases are linked to potassium channel mutation which has potentially important therapeutic consequences leading to transfer some patients from insulin therapy to sulfonylureas.

### B. Methods for the molecular diagnosis of the 6q2-4 anomalies associated with "transient" neonatal diabetes

The uniparental disomy of the chromosome 6 can be evidenced by the analysis of polymorphic markers present on the chromosome 6; meiotic segregation of the chromosomes can be determined by comparing the allelic profiles of polymorphic markers in the child and his parents. Usually, a total uniparental disomy of the chromosome 6 is evidenced, but partial one can also be found. This is why markers close to the region of interest (6q24) should be chosen. Chromosome 6 duplications can also be evidenced by that technique (Figures 2 and 3) [13]. The use of Polymerase Chain Reaction (PCR) after digestion of the DNA with a methylation sensitive enzyme permits detection of methylation defects (Figure 4) [15].

Whatever the technique, the presence of a chromosome 6q anomaly predicts a "transient" form of the disease. Its absence, however, does not rule out this form of neonatal diabetes mellitus.

## V. Treatment

### A. Therapeutic consequences in the case of potassium channel mutation

The K_ATP _channels have a central role in cell response to metabolic changes in many organs and especially in pancreatic insulin secreting cell. The advances in the comprehension of the physiological function of these channels, and in particular of the Kir6.2 and SUR1 subunits, has found a major clinical application for patients having permanent neonatal diabetes due to a *KCNJ11 *or a *ABCC8 *mutation. The transfer from insulin injections to oral glibenclamide therapy seems highly effective for most patients and safe [21,47]. This illuminates how the molecular understanding of some monogenic form of diabetes may lead to an unexpected change of the treatment in children. This is a spectacular example of how the pharmacogenomic approach improves in a tremendous way the quality of life of the young diabetic patients. In France and some others countries, the transfer of the patients from insulin to sulfonylureas should be made within the legal rules of the country, most often in the context of clinical trials approved by the Health Authorities, as the sulfonylureas are not licensed (even contra-indicated in same countries) to be used in children. These legal aspects of the treatment should not be under estimated due to potential deleterious side effects of the sulfonylureas.

### B. Management of the treatment of this very early onset type of diabetes, in the neonatal period

Insulin therapy is crucial in NDM to obtain satisfactory weight gain and growth in newborns with intra-uterine growth retardation. In some cases, glucose and caloric deprivation has been instituted in newborns in the face of hyperglycemia to avoid insulin therapy. This leads to further difficulties in weight gain. In fact, high caloric intake should be maintained in these newborns and insulin therapy given. Although pediatricians face numerous difficulties in managing insulin therapy in the newborn period, very few data are available on the methods of insulin delivery in neonatal diabetes. In infants with transient neonatal diabetes mellitus, control of the blood glucose concentration can be attained with ultralente insulin treatment, without any episodes of hypoglycemia [48]. These authors recommended subcutaneous injection of ultralente insulin, rather than lente or isophane (NPH) insulin, to avoid hypoglycemia during the treatment of transient neonatal diabetes mellitus. This has not been our experience. Multiple injections of regular insulin are sometimes difficult to manage. Short acting insulins, both human and analogue, are best avoided except when using intravenous soluble insulin infusions to initially stabilize the infant. However in the UK, isophane insulin on a once daily basis has afforded reasonable control. Potentially the insulin analogue (insulin glargine) with its very steady, flat pharmacokinetic profile might prove useful in this condition, although there is currently no license for children of this age.

In some centers in France, we have chosen the continuous subcutaneous insulin infusion (CSII) in all the cases of neonatal diabetes requiring subcutaneous insulin therapy for more than 15 days. We report here our experience in the five cases where initiation of the CSII was done in our departments. Four of the cases were TNDM (follow-up 7 months to 10 years). The practical aspects of the treatment were as follows. During the first days, the daily dose requirement was evaluated with intravenous insulin and glucose infusions. When good glycemic control was obtained, CSII therapy was started. Insulin was diluted to 4 to 10 units per milliliter. Insulin strategy to start CSII depended on the feeding conditions. Under enteral continuous feeding, 100% of the total daily dose was administered as basal rate. Under bottle feeding, the basal rate represented 30% of the total daily dose and boluses 70%, with the same insulin dose before each meal (number of meals 8, then 7, then 6). Blood glucose was monitored every 3 to 4 hours. Basal rate was adjusted on the night blood glucose measurements and boluses on the post-prandial ones. CSII therapy was started between day 7 and 55, one to 13 days after initiation of insulin therapy. During the first month of CSII therapy (dose at day 15 of CSII: 0.3 to 1 unit per kg/day), good glycemic control was achieved on a mean number of 240 blood glucose measurements (5 patients). Mean blood glucose was 1.73 g/l, no severe hypoglycemia was noted, and the mean number of hypoglycemia episodes (blood glucose <0.6 g/l) was 4.2 per month. Similar excellent results were achieved for the rest of the CSII in both the TNDM and the PNDM cases. We did not observe any cutaneous side effects. We conclude that during the neonatal period, CSII therapy is safe, more physiological, more accurate and easier to manage than injections. CSII allows us to match the insulin requirements of a newborn. CSII requires management and supervision by a experienced team of physicians and nurses.

Realizing how difficult it is to take care of a child of this age with diabetes mellitus should prompt clinicians to transfer these children to specialized centers. Insulin therapy and high caloric intake are the basis of the treatment. Insulin pump may offer an interesting therapeutic tool in this age group in experienced hands.

## VI. Genetic counseling

The risk of recurrence is different according to the "transient" or permanent form of the disease and to the different molecular mechanisms identified.

### Anomalies in the chromosome 6q region: a disease linked to imprinting

In the case of uniparental disomy, none of the allele of the mother is found in the proband, the risk of recurrence does not exist in theory and there is no transmission by the child. Partial uniparental disomy have been described, linked to a post-zygotic mechanism. The recurrence risk is probably weak or close to zero, as the anomaly is post-zygotic.

If partial duplication is present in the propositus, there is a risk of disease recurrence in the family and parents have to be tested. Carriers of the duplication have a 50% risk of transmitting the defect. If it is a *de novo* mutation, there is *a priori* no risk of recurrence, except if germinal mosaicism exists (not described so far). Concerning the risk of transmission, fathers will transmit both the genetic defect and the disease. Half of the children will have the disease, which will be present equally in boys and girls. If the mother has the anomaly, her children will not have the disease but the male offspring will eventually pass on the disease.

Imprinting anomaly: the logic says that the mother should pass it on (imprinting relaxation) but so far no familial case is known and the risk of transmission is unknown. Indeed, the cause of the imprinting relaxation is not identified and the identified children with this anomaly are too young to procreate.

### Mendelien inheritance

Recurrence risk is 25% in the recessive autosomal disorders (*EIF2AK*, *Glis3*, *PTF1A*, and *PDX1 *genes). IPEX syndrome is an X linked disorder.

The mutation in the genes encoding the potassium channel subunits are transmitted in the heterozygous state in a dominant way. Moreover, transmission by germinal mosaicism has here been described.

## VII. Prognosis

In the neonatal period, the prognosis is linked to the severity of the disease, the degree of dehydration and acidosis, as well the rapidity with which the disease is recognized and treated. In the following period, the prognosis is determined by the associated malformations and lesions. For example, in the case of potassium channel anomalies, neuropsychological and neuromuscular disturbances can be present. Finally, the prognosis rely on the metabolic control, as in all the forms of diabetes mellitus, which will determine the timing of appearance of the long standing diabetes complications.

## VIII. Conclusions, unresolved questions

Neonatal diabetes is a rare condition. However, it is probably of great relevance to our understanding of the cause of type 2 diabetes within the general population. We believe these rare single gene disorders are natural models for identifying new genes with possible relevance to type 2 diabetes. As discussed, the *IPF-1 *mutation is important in MODY 4 and in some familial forms of early onset type 2 diabetes and the SUR1 mutation plays a role in newborn diabetes as well as in type 2 diabetes in young adults. We hope that elucidating the etiology of other forms of neonatal diabetes will provide information on normal pancreatic development and the basis of the pathology underlying pancreatic dysfunction.
